# Inaugural editorial

**DOI:** 10.1007/s44154-021-00001-6

**Published:** 2021-08-18

**Authors:** Zhen-Sheng Kang, Jian-Kang Zhu

**Affiliations:** 1 State Key Laboratory of Crop Stress Biology for Arid Areas and College of Plant Protection, Northwest A&F University, Yangling, Shaanxi 712100 China; 2 Shanghai Center for Plant Stress Biology and Center for Excellence in Molecular Plant Sciences, Chinese Academy of Sciences, Shanghai, 201602 China

We are pleased to invite you to join us in celebrating the birth of a new life sciences academic journal, *Stress Biology*. *Stress Biology* was launched this year as an open access journal published by Springer Nature in partnership with Northwest A&F University of China. We plan to have the journal indexed by SCIE in 2024 and aim to obtain its first impact factor of no less than 5.0 in 2024.

All organisms in nature, including plants, animals and microbes, have to struggle with a variety of biotic (pathogens and pests) and abiotic (drought, salinity, heat, and cold etc.) stresses in order to survive and propagate. During the long history of evolution, these organisms have developed various strategies and mechanisms to cope with environmental stresses. Nevertheless, severe stresses are still the main challenges limiting the growth, health and productivity of plants and animals. Plants, animals, and microbes and their interactions are the vital parts of the ecology we depend on, and some of them such as crop plants, livestock and poultry provide the food, fiber and shelter for us. Global climate change brings with it more frequent extreme weathers, leading to outbreaks of diseases and pests, and severe drought and floods, heat waves and bitter cold, and causing great threats to agricultural production and food security. Therefore, understanding the mechanisms of stress resistance and developing technologies for improving the stress resistance of plants, animals and useful microbes is critical for sustainable agriculture. The mission of the *Stress Biology* journal is to promote stress biology research for the sake of agricultural sustainability and human well-being.

*Stress Biology* aims to be a leading international journal that is vigorously peer-reviewed and publishes important results of stress biology research. The journal publishes original, high quality research of all aspects of stress biology, including but not limited to work that (1) provides fundamental insights into the understanding of responses of plants, microorganisms and animals to abiotic and biotic stresses; (2) elucidates the mechanisms underlying the adaptation and resistance of plants, microorganisms and animals to biotic and abiotic stresses; and (3) uses biotechnological and other strategies to improve the resistance of plants, microorganisms and animals to abiotic and biotic stresses. Topics covered by the journal include: physiology, molecular biology, cell biology, microbiology, pathology, ecology, genomics, epigenetics, gene editing, stress resistance breeding, biotechnology, and so on. Article types include: original paper, short communications, reviews, highlights, letters to the editor, editorials.

*Stress Biology* will have all the good characteristics of a Springer Nature journal, including straightforward initial submission, fast and fair review with constructive comments, rapid online publishing prior to copyediting and typesetting, high-quality production, etc. Furthermore, as an open access journal, all contents will be freely available to researchers worldwide through the Springer Nature platform. The journal will cover article processing charge for authors during the period from 2021 to 2023.

The high-quality reviews and research articles published in this inaugural issue of *Stress Biology* cover a wide range of research topics in the field of stress biology and showcase the goals and scope of our journal: a high-quality open access journal with a broad scope for communicating various discoveries in stress biology that contribute to the development of sustainable agriculture worldwide.

We sincerely thank all of the authors who have contributed papers to this inaugural issue. We also would like to thank all of our editorial board members and the reviewers, as well as members of our editorial and production teams, for their support. Our joint efforts have now culminated in the successful launch of this new journal of our own research community! We look forward to working together with you all to grow this promising journal so that your important research discovery can have the great impact and benefits to society it deserves. Come and publish in *Stress Biology*!

**Editors-in-Chief**

**Zhen-Sheng Kang:** Academician of the Chinese Academy of Engineering, and Director of the State Key Laboratory of Crop Stress Biology for Arid Areas of China

**Jian-Kang Zhu:** Member of the US National Academy of Sciences, and Director of the Shanghai Center for Plant Stress Biology, Chinese Academy of Sciences

**Associate Editors**

**Jin-Rong Xu:** Professor of Purdue University, USA

**Huazhong Shi:** Associate professor of Texas Tech University, USA

**Executive editor**

**Qiao-Chun Wang:** Professor of Northwest A&F University, China

